# Impact of the COVID-19 Pandemic on Acute Upper Gastrointestinal Bleeding in Xingtai City

**DOI:** 10.1155/2021/5586030

**Published:** 2021-03-06

**Authors:** Zhihui Duan, Qiong Duan, Kun Liu, Xiaochong Zhang, Shengyun Zhou, on behalf of Xingtai Society of Digestive Endoscopy

**Affiliations:** ^1^Department of Endoscopy, Xingtai People's Hospital, Xingtai, 054000 Hebei Province, China; ^2^Department of Gastroenterology, Xingtai People's Hospital, Xingtai, 054000 Hebei Province, China; ^3^Department of Radiology, Xingtai Fifth Hospital, Xingtai, 054000 Hebei Province, China; ^4^Institute of Cancer Control, Xingtai People's Hospital, Xingtai, 054000 Hebei Province, China; ^5^Xingtai People's Hospital, China

## Abstract

**Background and Aims:**

The coronavirus disease 2019 (COVID-19) has severely impacted the daily practice of gastrointestinal endoscopy worldwide. Most endoscopy centers in China were shut down in late January 2020. We investigated the impact of the shutdown on acute upper gastrointestinal bleeding (AUGIB) events in Xingtai City, Hebei Province, China.

**Methods:**

A web-based survey collected information on gastroscopy workload and AUGIB events. The study period was from 4 weeks before to 4 weeks after lockdown initiation in Xingtai City. Fourteen public gastrointestinal endoscopy centers performing emergency endoscopies were contacted via e-mail to collect weekly emergency gastroscopy volumes and the number of AUGIB events. AUGIB was defined as recent melena, hematemesis, or both, with an endoscopically visible source of bleeding.

**Results:**

Twelve (85.7%) of the 14 surveyed gastrointestinal endoscopy centers in the city- and county-level hospitals responded. Altogether, 4,045 and 1,077 gastroscopy procedures were performed 4 weeks before and after lockdown initiation (73.4% reduction), respectively. Peptic ulcer-related AUGIB and variceal AUGIB events showed a 58.5% and 52.9% decline, respectively, compared with pre-COVID-19 data. Although the absolute number of AUGIB events decreased during the pandemic (from 149 to 66), the likelihood of detecting AUGIB during gastroscopy increased (3.68% (pre-COVID-19 period) versus 6.13% (COVID-19 period); *P* < 0.05).

**Conclusion:**

The COVID-19 pandemic resulted in a considerable reduction in gastroscopy workload and AUGIB events; however, the likelihood of detecting AUGIB increased significantly during gastroscopies.

## 1. Introduction

The coronavirus disease 2019 (COVID-19), caused by the severe acute respiratory syndrome coronavirus 2 (SARS-CoV-2), rapidly spread from Wuhan to other provinces in China and worldwide [1, 2]. To curb its spread, a lockdown in Wuhan was initiated on January 23, 2020 [3], which largely inhibited population migration from Wuhan. On January 27, 2020, the first confirmed case was reported in Xingtai City, Hebei Province [4]. Subsequently, many gastrointestinal endoscopy (GIE) centers were shut down for a mean period of 1 month after the Spring Festival Holiday on February 1, 2020 [5]. The World Health Organization (WHO) declared COVID-19 as a global pandemic on March 11, 2020 [6]. Currently, this global pandemic has affected the daily practice of GIE and will continue to do so in the future. To prevent SARS-CoV-2 infection during endoscopic procedures, the international GIE societies have published several guidelines and position statements focused on endoscopy during the pandemic [7–9]. Sagami et al. recently confirmed that upper GIE is an aerosol-generating procedure [10]. Thus, as recommended by the established guidelines and statements, the deferment of elective endoscopies should be considered during the pandemic, and urgent endoscopies, such as those for acute gastrointestinal bleeding (GIB), should be performed by well-trained staff to minimize the risk of disease transmission [7–9].

The lockdown has resulted in a large decline in acute upper GIB (AUGIB) events and endoscopy volumes [11, 12]. In Austria, the percentage of AUGIB events reduced by 40.7% during the 3 weeks of lockdown compared to the 3 weeks before lockdown [11]. Few studies on the impact of COVID-19 on AUGIB have been reported in China. We aimed to investigate how the lockdown might have influenced the UGIB events in Xingtai City, which has 7.39 million permanent residents and where the COVID-19 pandemic curve has been relatively flattened.

## 2. Methods

### 2.1. Study Design

In this retrospective study, we developed a web-based survey using an Excel worksheet, which was sent to 14 GIE centers in Xingtai City to collect information on the weekly number of gastroscopy procedures performed in a specific center, as well as UGIB events and their cause from January 4, 2020, to February 28, 2020. We chose all the major city- and county-level GIE centers in Xingtai that responded to our survey successfully; those that responded with incomplete data were excluded. All the participating centers used a uniform tool to report the gastroscopy findings. The shutdown of GIE centers in Xingtai City started on February 1, 2020, and all the surveyed centers followed this policy. This study was approved by the Medical Ethical Review Board of Xingtai People's Hospital. Patients presenting with signs and symptoms of AUGIB (melena, hematemesis, or both, and decreased hemoglobin levels) were included in the study. UGIB are all those that also had an endoscopic finding explaining the symptoms. The data on the cause of UGIB was collected and analyzed. All responses were analyzed anonymously, so written informed consent was waived.

### 2.2. Inclusion Criteria for Gastrointestinal Endoscopy Centers and Patients

Adult patients presenting with AUGIB were admitted to the city- or county-level GIE centers in Xingtai City from January 4, 2020, to February 28, 2020. AUGIB was defined as evidence of hematemesis, coffee-ground emesis, or melena, with a 2 g/dL decline in hemoglobin. AUGIB was characterized according to its etiology and anatomical location. Patients with incomplete data or incomplete gastroscopy examination were excluded.

### 2.3. Statistical Analysis

We used SPSS version 24.0 for statistical analysis and drawing. Qualitative data were presented as numbers and percentages and were compared by the chi-squared test or Fisher's exact test. A two-sided *P* value of <0.05 was considered statistically significant.

## 3. Results

### 3.1. Endoscopy Results

**Figure 1:**
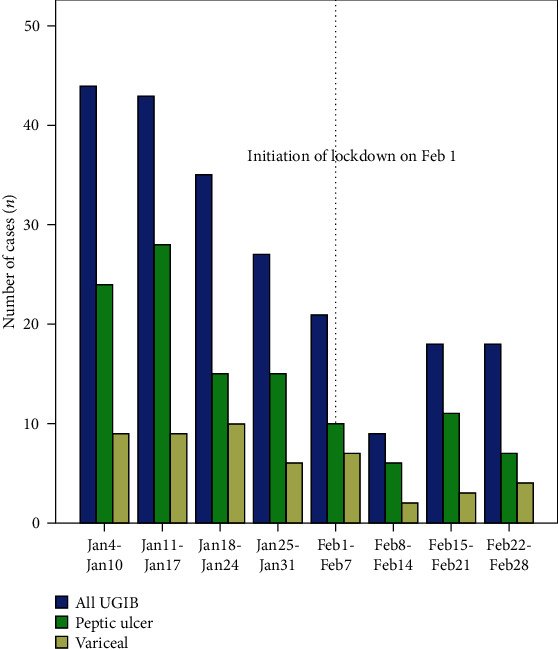
Decrease in the number of acute upper gastrointestinal bleeding events in Xingtai City, Hebei Province, China, before and after the start of lockdown during the coronavirus disease 2019 (COVID-19) pandemic. The weekly gastroscopy volumes for different bleeding causes are shown. The lockdown was initiated on February 1, 2020. Blue bars: total weekly number of UGIB events; green bars: peptic ulcer bleeding; yellow bars: variceal bleeding. Dashed lines correspond to the initiation of lockdown. Abbreviations: COVID-19: coronavirus disease 2019; UGIB: upper gastrointestinal bleeding.

**Figure 2:**
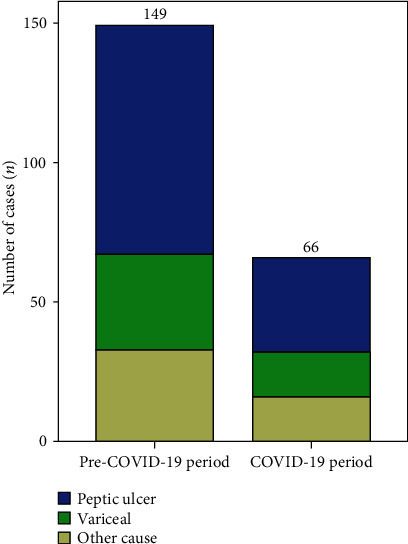
The decline in the absolute number of endoscopic findings of acute upper gastrointestinal bleeding with different causes. Comparison of the percentages of detected causes of UGIB between the pre-COVID-19 and COVID-19 periods. Abbreviations: COVID-19: coronavirus disease 2019; UGIB: upper gastrointestinal bleeding.

We contacted 14 city- and county-level public hospitals in Xingtai City, Hebei Province, China. Twelve (85.7%) hospitals responded to our survey and provided the number of weekly esophagogastroduodenoscopy (EGD) procedures and AUGIB events, including variceal and peptic ulcer bleeding. Data on the number of EGD procedures performed in weeks 1-8 are shown in Figure 1. During the study period, a dramatic decrease in the number of EGD procedures was observed since the COVID-19 outbreak. Overall, 4,045 EGD procedures were performed for AUGIB during the 4 weeks before the lockdown, which was higher than the number of EGD procedures performed during the 4 weeks after the lockdown (*n* = 1,077), with a 55.7% decline (from 149 to 66) in UGIB (*P* < 0.001) (Figure 2). During the 4-week lockdown period, the reduction rates of peptic and variceal UGIB were 58.5% (from 82 to 34) and 52.9% (from 34 to 16), respectively. Despite this, UGIB was diagnosed significantly more often in patients who had undergone EGD during the COVID-19 period compared to those who had undergone EGD during the pre-COVID-19 period (6.13% vs. 3.68%, *P* < 0.05, Table 1). During the COVID-19 period, peptic ulcer bleeding remained the main cause of UGIB (51.5%), followed by variceal bleeding (24.2%). The proportion of peptic ulcer disease (PUD) (from 55.0% to 51.5%) and variceal bleeding (from 22.8% to 24.2%) in UGIB remained almost equal between the two periods (Figure 2 and Table 2).

### 3.2. Trends in Endoscopy Volume in Xingtai People's Hospital Single Center

**Figure 3:**
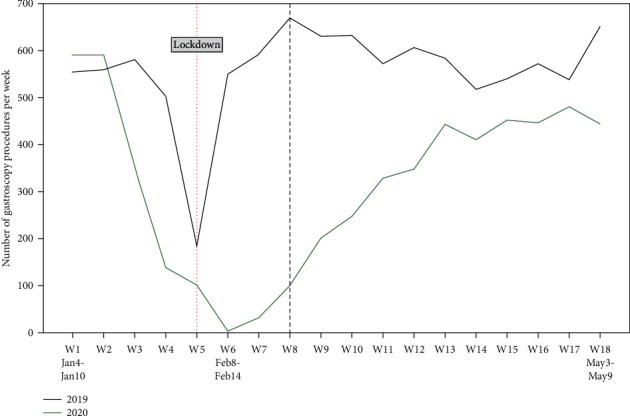
Number of gastroscopy procedures performed weekly in Xingtai People's Hospital between January 4 and May 9 in both years 2019 and 2020. Dashed lines correspond to the initiation and partial lifting of the lockdown.

Figure 3 shows the gastroscopy volumes from January 4, 2020, to May 9, 2020, in Xingtai People's Hospital; a considerable decline was seen in the absolute volumes of gastroscopies performed during the lockdown (the period between the dashed vertical lines). After the partial relaxation of the restrictions during the lockdown on March 1, 2020, the gastroscopy volumes gradually increased; however, on May 9, the volumes remained lower than those observed in the same period in 2019.

## 4. Discussion

The pandemic lockdown implemented by the Chinese government led to a massive reduction in gastroscopy volumes and UGIB events. Our data on the number of UGIB events in Xingtai City suggest that most bleeding cases must have been reported by the large public city- and county-level GIE centers. However, patients undergoing gastroscopy during the lockdown period were more likely to be diagnosed with UGIB.

The volume of gastroscopy procedures showed a significant decline since the start of the lockdown, which is consistent with the findings of previous studies [11–14]. This can be partially explained by the established guidelines' recommendation that elective endoscopic procedures should be delayed or canceled to minimize the risk of COVID-19 transmission [7–9]. Besides this, the public had concerns about seeking help during the lockdown. As expected, the number of UGIB events decreased from 149 to 66, showing a 55.7% reduction rate, which is similar to the rate reported in Austria (40.7%) [11]. The reasons for the decline in the UGIB events are currently unclear. One possible reason is that people are reluctant to seek medical care due to the fear of contracting COVID-19. Moreover, public transportation was suspended during the lockdown, thereby limiting patients' access to healthcare services. Furthermore, the screening procedures before endoscopy, such as chest computed tomography, temperature taking, use of epidemiological questionnaires, and COVID-19 polymerase chain reaction tests, when necessary, were very strict. Patients with negative screening results could undergo gastroscopy. Patients with relatively mild UGIB symptoms may have chosen not to visit GIE centers. Moreover, gastroenterologists might choose to conservatively treat patients with UGIB to minimize the transmission risk for both patients and personnel. Over the same observation period, the number of variceal bleeding cases decreased by 52.9% (from 34 to 16) in our study, which was higher compared to that reported in Austria (10.8%; from 37 to 33) [11]. One interesting finding in our study was the increase in the percentage of patients diagnosed with UGIB during gastroscopies during the pandemic (from 3.68% to 6.13%). This echoed the findings by Lau et al., who reported that the percentage of patients receiving emergent endoscopies for UGIB during the pandemic increased from 30% to 38.9% (*P* < 0.001) [15].

In the survey conducted in Austria [11], variceal bleeding accounted for <10% of UGIB events and was not influenced by lockdown restrictions. However, in our study, variceal bleeding events constituted 24.2% of all UGIB events occurring during the pandemic, which was higher than 10%, but it was not affected by the lockdown restrictions (from 22.8% to 24.24%, *P* > 0.05). One possible reason might be that the patients with severe variceal bleeding requiring urgent interventions ultimately underwent EGD, whereas those with peptic ulcer bleeding who had less severe symptoms were more likely to be conservatively managed [14]. In a multicenter study, variceal upper gastrointestinal bleeding accounted for 14.6% during the COVID-19 pandemic, with 17% before the COVID-19 pandemic [16]. Certainly, the small sample size might have also influenced the result. In Northern Italy, the most common cause of UGIB was peptic ulcer disease [17].

This study has several limitations. First, the study's retrospective and multicenter design may have led to inconsistencies in determining UGIB cases. Second, data for the comparison of the gastroscopy volumes between the years 2019 and 2020 were only available from a single center, specifically Xingtai People's Hospital, which is one of the largest GIE centers in Xingtai City. Third, our study period, which was 4 weeks before and 4 weeks after the start of lockdown, was somewhat short. Fourth, the pandemic situation varies among different areas; thus, our findings only reflect immediate outcomes and cannot be generalized to other areas with different pandemic situations. A national survey with a larger sample size needs to be conducted in the future to clarify the impact of COVID-19 on endoscopy activity and AUGIB events. Fifth, we did not investigate the mortality rate of upper GIB because we did not collect the relevant data. Similarly, Schmiderer et al. [11] also did not investigate the mortality due to UGIB in the pandemic. However, one previous study has addressed the question. As Kim et al. [14] reported, inpatient mortality of GIB was similar between the pre-COVID-19 and COVID-19 periods.

In conclusion, the 4-week rigorous national lockdown significantly reduced the absolute gastroscopy volumes and AUGIB events in Xingtai City, Hebei Province, which may have a long-lasting impact on the practice of gastroscopy. We believe that our data will help GIE centers in managing their routine activities and AUGIB cases during and after the pandemic.

## Figures and Tables

**Table 1:** Details of the gastroscopy procedures performed, categorized by the cause of UGIB.

Details	Pre-COVID-19 period (4-Jan to 31-Jan) (*N* of EGD = 4,045)	COVID period-19 (1-Feb to 28-Feb) (*N* of EGD = 1,077)	*P* value
All UGIB events (*n* (%))	149 (3.68%)	66 (6.13%)	0.016
Peptic ulcer UGIB (*n* (%))	82 (2.03%)	34 (3.16%)	0.027
Variceal UGIB (*n* (%))	34 (0.84%)	16 (1.49%)	0.056

*P* values were compared between the two groups. A significant level of *P* < 0.05 was used. Abbreviations: COVID-19: coronavirus disease 2019; UGIB: upper gastrointestinal bleeding; EGD: esophagogastroduodenoscopy.

**Table 2:** The proportion of peptic ulcer and variceal bleeding events in the pre-COVID-19 and COVID-19 periods.

Details	Pre-COVID-19 period (4-Jan to 31-Jan) (*N* of UGIB = 149)	COVID-19 period (1-Feb to 28-Feb) (*N* of UGIB = 66)	*P* value
Peptic ulcer UGIB (*n* (%))	82 (55.03%)	34 (51.52%)	0.633
Variceal UGIB (*n* (%))	34 (22.82%)	16 (24.24%)	0.821

*P* values were compared between the two groups. A significant level of *P* < 0.05 was used. Abbreviations: COVID-19: coronavirus disease 2019; UGIB: upper gastrointestinal bleeding; EGD: esophagogastroduodenoscopy.

## Data Availability

Data are available on reasonable request through 15131988129@163.com.

## Abbreviations

COVID-19: Coronavirus disease 2019
AUGIB: Acute upper gastrointestinal bleeding
GIE: Gastrointestinal endoscopy
WHO: World Health Organization
GIB: Gastrointestinal bleeding
EGD: Esophagogastroduodenoscope
PUD: Peptic ulcer disease.

## Supplementary Materials

Supplementary Materials Supplementary Material contains the designed questionnaire which was sent to investigate endoscopy centers.
