# Supplementary figures and images for: Impact of the COVID-19 Pandemic on Acute Upper Gastrointestinal Bleeding in Xingtai City
Source: Gastroenterol Res Pract. 2021 Mar 6;2021:5586030. doi: 10.1155/2021/5586030 (PMC7938257; doi:10.1155/2021/5586030)

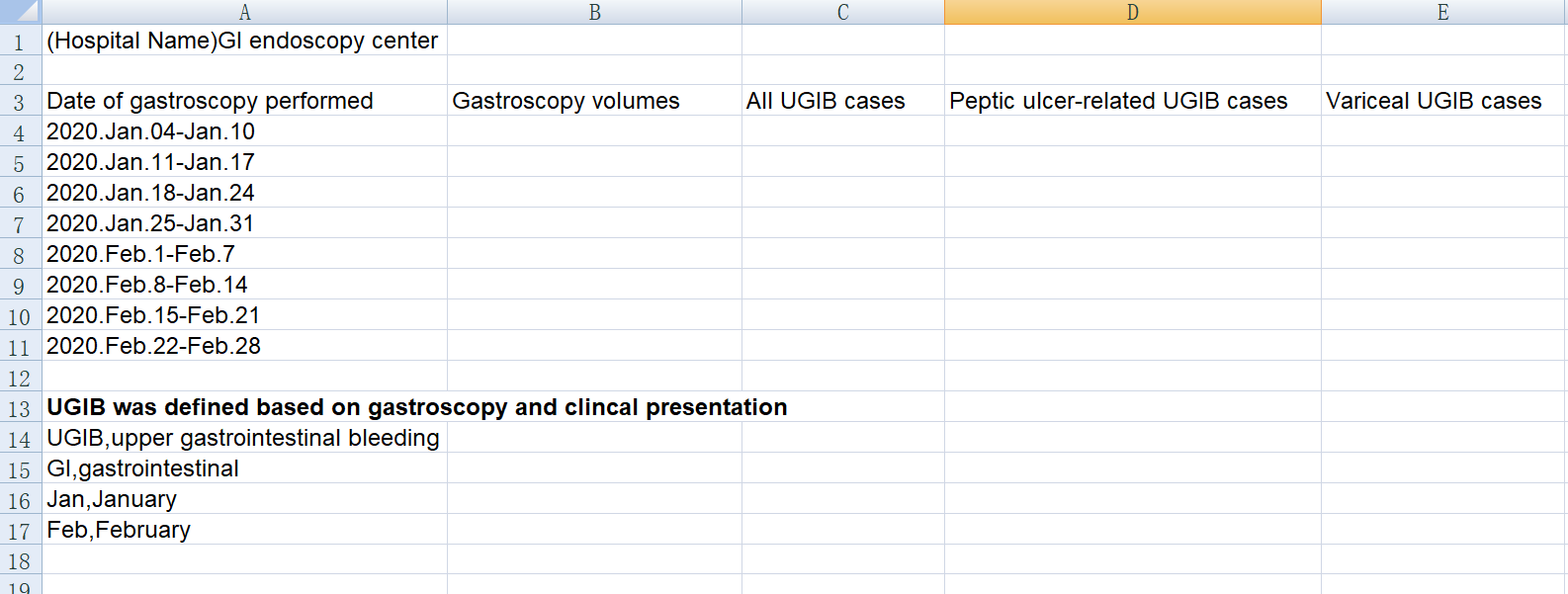

Supplement: Supplementary Materials — Supplementary Material contains the designed questionnaire which was sent to investigate endoscopy centers. [file 5586030.f1.docx]
